# An effective antibiofilm strategy based on bacteriophages armed with silver nanoparticles

**DOI:** 10.1038/s41598-024-59866-y

**Published:** 2024-04-20

**Authors:** Mateusz Szymczak, Jarosław A. Pankowski, Agnieszka Kwiatek, Bartłomiej Grygorcewicz, Joanna Karczewska-Golec, Kamila Sadowska, Piotr Golec

**Affiliations:** 1 Department of Molecular Virology, Faculty of Biology, Institute of Microbiology, University of Warsaw, Miecznikowa 1, 02-096 Warsaw, Poland; 2 Dioscuri Centre for Physics and Chemistry of Bacteria, Institute of Physical Chemistry, Polish Academy of Sciences, Kasprzaka 44/52, 01-224 Warsaw, Poland; 3 Nalecz Institute of Biocybernetics and Biomedical Engineering, Polish Academy of Sciences, Ks. Trojdena 4, 02-109 Warsaw, Poland

**Keywords:** Bacteriophages, Nanoparticles

## Abstract

The emerging antibiotic resistance in pathogenic bacteria is a key problem in modern medicine that has led to a search for novel therapeutic strategies. A potential approach for managing such bacteria involves the use of their natural killers, namely lytic bacteriophages. Another effective method involves the use of metal nanoparticles with antimicrobial properties. However, the use of lytic phages armed with nanoparticles as an effective antimicrobial strategy, particularly with respect to biofilms, remains unexplored. Here, we show that T7 phages armed with silver nanoparticles exhibit greater efficacy in terms of controlling bacterial biofilm, compared with phages or nanoparticles alone. We initially identified a novel silver nanoparticle-binding peptide, then constructed T7 phages that successfully displayed the peptide on the outer surface of the viral head. These recombinant, AgNP-binding phages could effectively eradicate bacterial biofilm, even when used at low concentrations. Additionally, when used at concentrations that could eradicate bacterial biofilm, T7 phages armed with silver nanoparticles were not toxic to eukaryotic cells. Our results show that the novel combination of lytic phages with phage-bound silver nanoparticles is an effective, synergistic and safe strategy for the treatment of bacterial biofilms.

## Introduction

The increasing prevalence of antibiotic-resistant microorganisms is becoming a fundamental threat to public health^1^. Multidrug-resistant (MDR) bacteria are defined as organisms that are not susceptible to at least 1 agent from ≥ 3 categories of antimicrobial compounds^2^. They often cause severe skin diseases; adversely affect the reproductive organs, urinary tract, heart, and lungs; and cannot be easily treated with standard antibiotics^3^. Therefore, substantial efforts are underway to identify alternative methods for controlling MDR infections^4^.

MDR bacteria capable of forming biofilm structures can be particularly dangerous because of their ability to cause chronic and recurring infections^5^. Biofilms can also facilitate horizontal gene transfer, thereby promoting the spread of antibiotic resistance genes^6^. Moreover, biofilm structure promotes the colonization of both human and animal bodies, as well as abiotic surfaces. These properties have led to an increasing number of infections associated with the use of biomaterials in human and veterinary medicine^7^.

Bacteriophages (phages) are commonly occurring viruses that infect bacteria. They have applications in controlling biofilm formation and treating bacterial infections^8^. A phage cocktail (i.e., a mixture of various phages) can significantly reduce biofilm growth on both food and abiotic surfaces, substantially aiding in disease prevention. For example, recent studies involving *Escherichia coli* and *Pseudomonas aeruginosa* showed that phages were able to prevent microbial colonization of beef products and endotracheal tubes, respectively, indicating that this method is effective for controlling virulent microbes^9,10^. Additionally, phages can cause the lysis of various *Salmonella* serovars and significantly reduce biofilm development on artificially inoculated dairy and poultry products, further reinforcing the notion that this method can be used in real-world settings^11^. Similarly, *Staphylococcus epidermidis* infection in a prosthetic knee has been successfully treated with phage therapy after debridement and implant retention surgery^12^.

The combination of phages with other treatment approaches (e.g. antibiotics) can enhance therapeutic effects, as demonstrated by the antimicrobial synergy between the temperate phage HK97 and ciprofloxacin during the eradication of *E. coli *in vitro^13^. The combination of another phage, PEV20, with ciprofloxacin was effective for controlling disease progression in mice with *P. aeruginosa* pulmonary infections^14^. Moreover, the combination of Sb-1 phage with oxacillin significantly reduced the emergence of methicillin resistance among isolates of *Staphylococcus aureus*^15^. These results demonstrate that the combined use of phages with other antimicrobial agents can effectively control the spread of MDR bacteria.

Metal nanoparticles are materials in the nanometer range that comprise a single metallic element or its oxide^16^. They have numerous applications in medicine and industry. Silver nanoparticles (AgNPs) are particularly intriguing because they have often been successfully used in antimicrobial therapy^17^. Studies have shown that AgNPs can effectively disrupt plasma membranes in Gram-negative bacteria by causing ion loss and inhibiting respiration^18^, cause the generation of reactive oxygen species in Gram-negative and Gram-positive bacteria^19^, and release silver ions upon dissolution that block active sites in enzymes in Gram-positive bacteria.^20^. Silver nanoparticles can be effective against MDR strains of *E. coli*^21^, *P. aeruginosa*^22^, *Klebsiella pneumoniae*^23^, *S. aureus*, *S. epidermidis,* and *Acinetobacter baumannii*^22,24^. Furthermore, they can synergize with antibiotics such as cefotaxime, ceftazidime, meropenem, ciprofloxacin, and gentamicin to eradicate *E. coli* and *K. pneumoniae*^25^. A combination of AgNPs and ampicillin demonstrated efficacy in terms of killing strains of *E. coli*, *S. aureus*, *K. pneumoniae,* and *P. aeruginosa*^26^. Silver nanoparticles alone were effective in controlling biofilm formation by species such as *E. coli*, *Enterococcus faecalis*, *Bacillus subtilis*, *S. aureus*, *Streptococcus mutans*, *S. typhimurium*, *P. aeruginosa,* and *K. pneumoniae*; AgNPs were also effective in controlling the formation of mixed-species biofilms^27–31^. Finally, AgNPs could control the formation of biofilms by the pathogenic yeast *Candida albicans*^27^. The combination of AgNPs with other antibiofilm agents often produces stronger effects^32^. Thus, AgNPs can be used as a component of broader antimicrobial therapy that prevents biofilm formation and facilitates recovery.

Here, we report that T7 phages armed with AgNPs exhibit superior antibiofilm efficacy compared with the use of either treatment alone. We first identified a novel peptide that binds AgNPs, then constructed recombinant T7 phages that displayed this peptide on their capsids (T7Ag-XII-AgNP bionanomaterial). In *E. coli* biofilm treatment assays, the bionanomaterial made of engineered T7Ag-XII phages and AgNPs was more effective than T7wt phages or AgNPs used separately or as a mixture, suggesting that it can be an effective alternative to antibiotics. Importantly, T7Ag-XII phages bound with AgNPs were not toxic to eukaryotic cells when low concentrations of AgNPs were used to develop the bionanomaterial.

## Results

### Identification of novel AgNP-binding peptides

**Figure 1:**
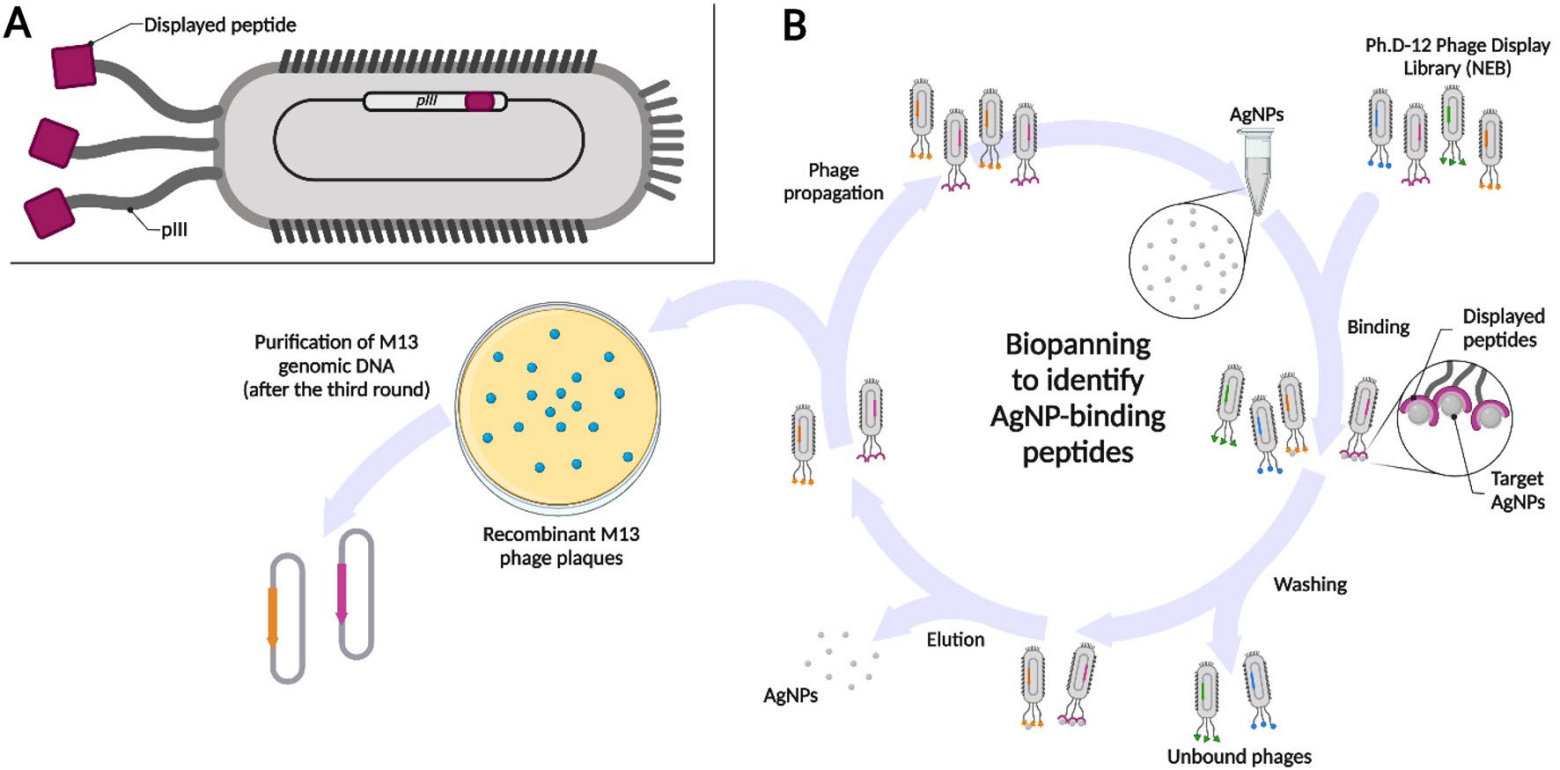
Schematic of a recombinant M13 bacteriophage and the biopanning procedure. (**A**): An M13 phage (with a nucleotide insert inside of the *pIII* gene) displaying a peptide within the pIII protein (purple). (**B**): The biopanning procedure enabled the isolation and identification of M13 phages that displayed specific AgNP-binding peptides. After three rounds of biopanning, the phages were propagated from separated plaques, and their genomic material was isolated for sequencing and identification of AgNP-binding peptides. Created with BioRender.com. *AgNP*, Silver nanoparticle; *NEB*, New England Biolabs.

Initially, we sought to identify novel peptides with AgNP-binding properties. Accordingly, we used the commercially available Ph.D-12 Phage Display Peptide Library (New England Biolabs), which involves peptide display within the pIII protein of the M13 phage (Fig. 1A). We used a commercially available silver nanopowder as a target material in the biopanning search procedure. Three rounds of biopanning (Fig. 1B) yielded recombinant M13 phages that displayed peptides with the desired AgNP-binding properties.

After 3 rounds of biopanning, 20 recombinant M13 phages were separately propagated and subjected to isolation of genomic DNA. Sequencing of the nucleotide inserts within the *pIII* gene in each recombinant phage resulted in the identification of seven individual AgNP-binding amino acid sequences (Table S1). While all identified peptides were expected to bind AgNPs, differences in values of pI and molecular weight (MW) of specific peptides may suggest their different efficiency in this process.

### Efficacy of AgNP binding by M13 phages

**Figure 2:**
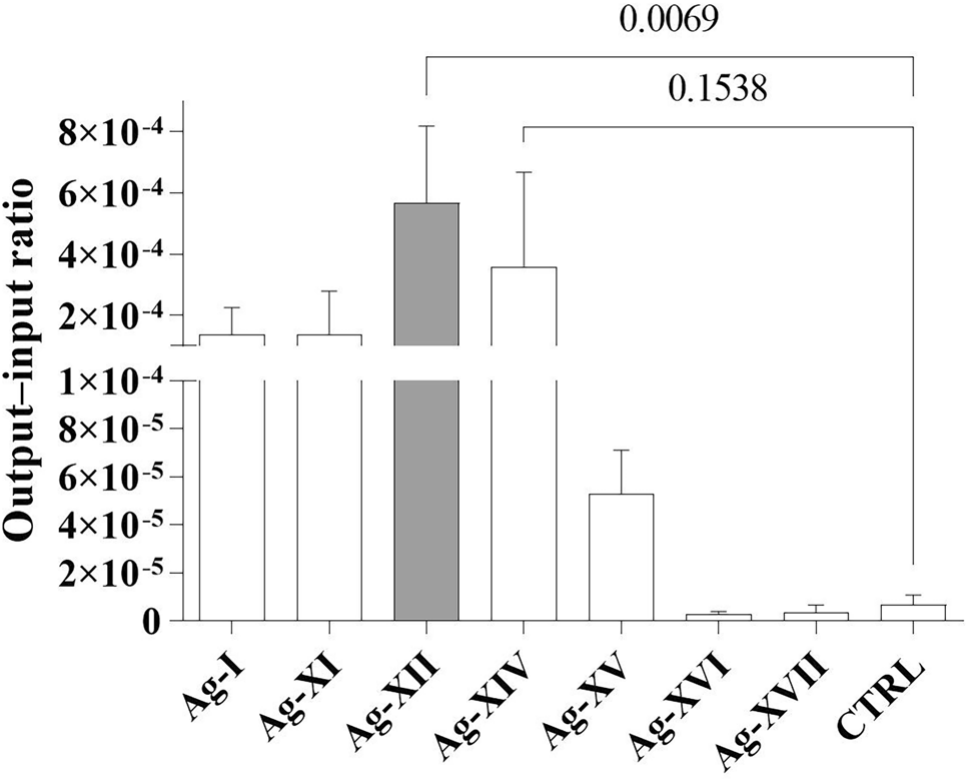
Efficacy of AgNP binding by peptides (listed in Table S1) displayed within the pIII protein on M13 phage virions. Efficacy was assessed in terms of the output–input ratio. “Output” comprised phages that bound to AgNPs, whereas “input” comprised the total number of phages used in the binding analysis. The gray bar indicates the highest efficacy of AgNP binding (by the Ag-XII peptide that comprises the RFEHPAVPRTEM sequence). Unmodified M13KE phage was used in the control experiment (CTRL). Data are shown as mean values from three experiments, and standard deviations are represented by error bars. For statistical analysis, a one-way ANOVA test was carried out.

Although the identified peptides were expected to bind to AgNPs, experimental confirmation of this binding was necessary to identify the best candidate for subsequent analyses. Therefore, we examined the AgNP-binding efficacies of the identified peptides (Fig. 2) using a binding efficiency assay that is based on the ratio of the output phage number (i.e. phages that bound to AgNPs in the AgNP-binding assay) to the input phage number (i.e. the total number of recombinant phages used in the AgNP-binding assay)^33^. We found that the Ag-XII peptide (RFEHPAVPRTEM) exhibited the best AgNP-binding efficacy. We noted that the difference in AgNP-binding efficacy between the Ag-XII and Ag-XIV (LPYSARTFFSEA) peptides was not significant; only the binding efficacy of Ag-XII differed significantly compared to the control (CTRL). Therefore, we chose only the Ag-XII peptide for further analyses.

### Construction of T7Ag-XII phages displaying AgNP-binding peptides

M13 phages are not lytic and are not used in therapeutic applications. An analysis of antimicrobial synergy between phages and AgNPs must be conducted using lytic phages that can display specific peptides on external proteins. For this analysis, we selected the T7 phage because its genome can be modified to display peptides, and T7-like phages often exhibit therapeutic potential^34,35^.

**Figure 3:**
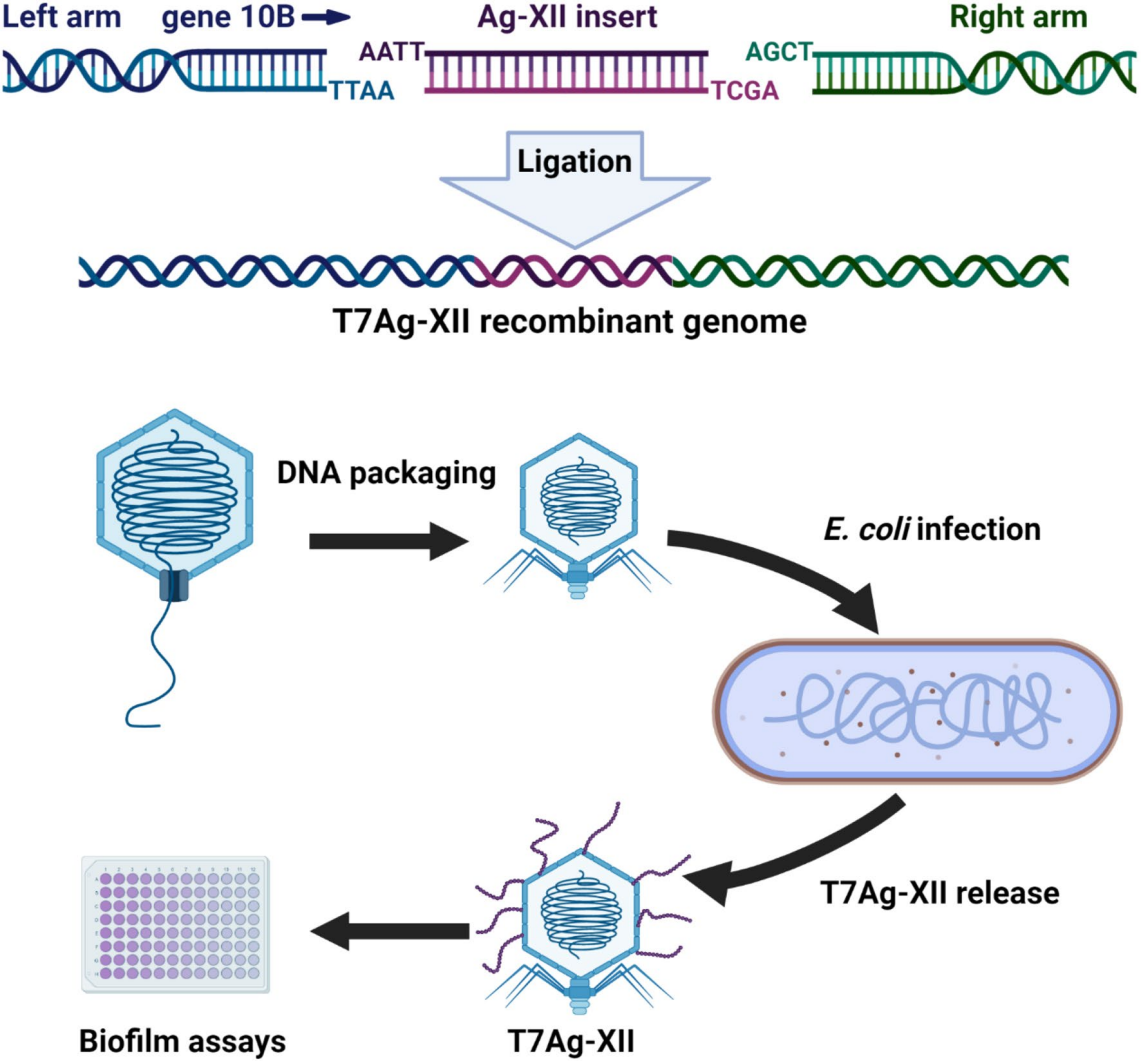
Schematic of the construction of recombinant T7Ag-XII phage. Synthesized oligonucleotides encoding the Ag-XII peptide were ligated to the left and right arms of the T7 genome. The resulting genomic construct was packaged into empty T7 capsids and used to infect *Escherichia coli* BLT5403. Subsequently, the recombinant T7Ag-XII phage was used in biofilm degradation assays. Created with BioRender.com.

The nucleotide sequence encoding the selected peptide was assembled from two single-stranded DNA oligonucleotides. Denaturation and annealing resulted in double-stranded DNA fragments with short single-stranded overhangs. These double-stranded DNA fragments allowed ligation to complementary regions within the arms of the T7 genome from the T7Select System. Next, the recombinant T7 genomic constructs were packaged into T7 phage capsids using the commercially available T7Select system (Fig. 3). The presence of the correct DNA insert in the T7 genome was confirmed by Sanger sequencing.

### Analysis of interactions between T7 phages and AgNPs

To date, combinations of phages and AgNPs have been analyzed for improved antibacterial activity mostly as a simple mixture of these agents. In our study, engineered T7Ag-XII phages bound to silver nanoparticles through the newly identified peptide were employed as an antibacterial bionanomaterial. Prior to conducting antibacterial assays, we explored the nature of interactions between T7Ag-XII and AgNPs in the bionanomaterial created.

**Figure 4:**
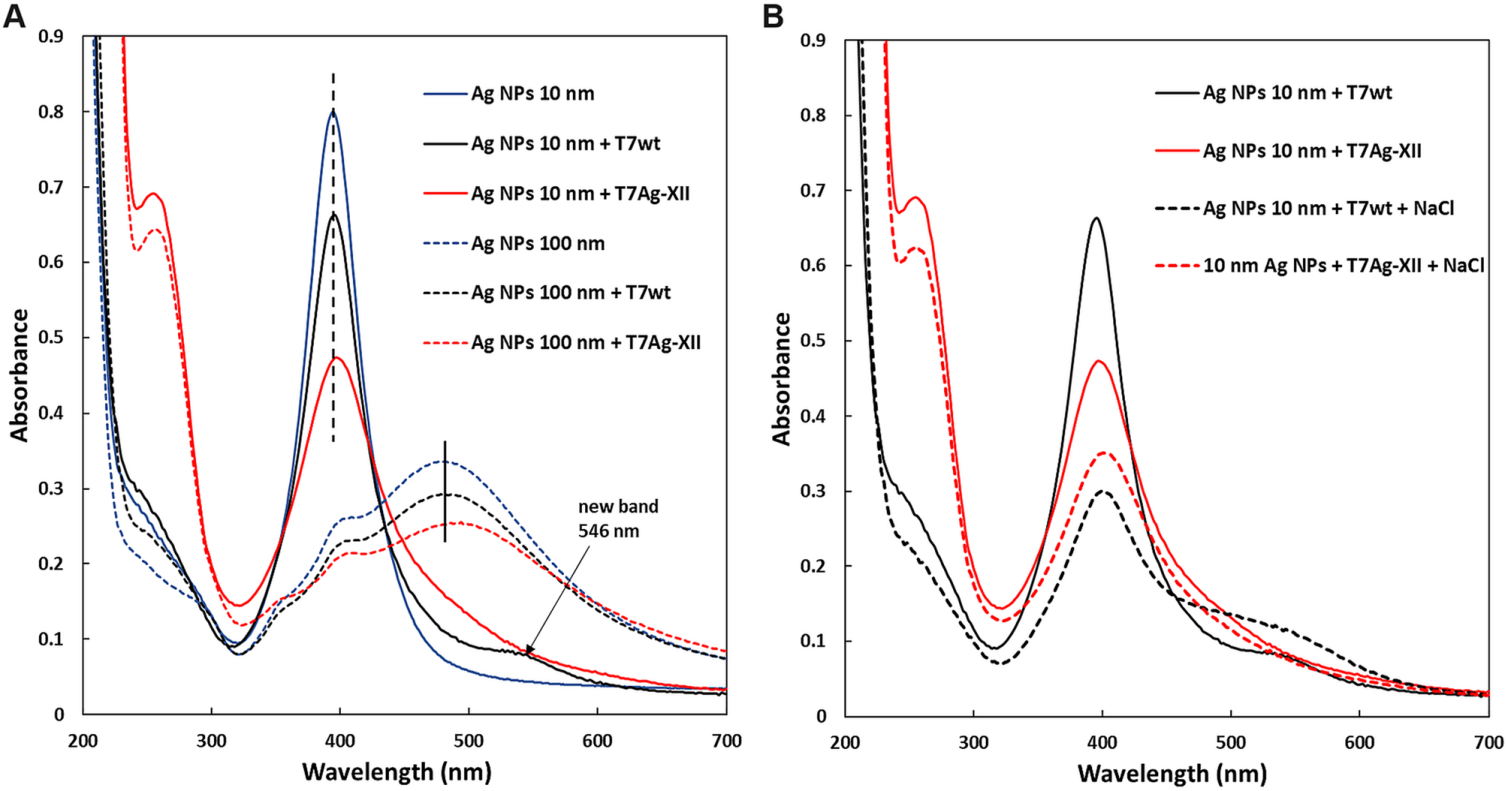
UV–Vis spectra of T7Ag-XII-AgNPs bionanomaterial, T7wt + AgNPs mixture, and T7Ag-XII, T7wt and AgNPs alone. UV–Vis spectra were recorded for AgNPs in the presence of T7wt and T7Ag-XII (100 µL of each AgNPs dispersion and 100 µL of T7wt or T7Ag-XII, each at a concentration of 1 × 10^9^ pfu/mL, were mixed and incubated for 30 min) (**A**). UV–Vis spectra of 10 nm AgNPs in the presence of T7wt or T7Ag-XII, each at a concentration of 1 × 10^9^ pfu/mL, after the addition of NaCl (**B**).

Silver nanoparticles typically display UV–Vis absorption maximum within the 400–550 nm range due to localized surface plasmon resonance (LSPR) phenomenon. Smaller nanospheres primarily absorb light with a peak near 400 nm, whereas larger spheres exhibit increased scattering, resulting in multiple bands that broaden and shift towards longer wavelengths. Previous research showed that UV–Vis spectroscopy results are sensitive to size, shape, concentration, agglomeration state, and refractive index near the nanoparticle surface (which varies with different capping agents)^36,37^. Therefore, we employed this technique to confirm that T7Ag-XII phages and AgNPs are indeed bound to each other and form a new bionanomaterial (Fig. 4A).

The UV–Vis spectra of the examined AgNPs (spherical, 10 and 100 nm) align with those of previous reports. The spectrum of spherical 10-nm AgNPs exhibited a sharp, well-defined band at 395 nm, while for larger, 100-nm AgNPs a broad, multiple band with a maximum at 485 nm developed. As depicted in Fig. 4A, the addition of either T7wt or T7Ag-XII into the dispersions of AgNPs of both sizes led to a decrease in peak intensity, with a more distinct effect observed after the addition of T7Ag-XII. A signal shift (to 402 and 500 nm for 10 and 100 nm AgNPs, respectively) and signal broadening were also observed. According to the Mie’s theory^38^, the observed absorbance in the metal nanoparticle spectra is correlated with scattering and the dielectric constant of the metal and its surroundings. If the ligand (in our case: T7Ag-XII) interacts with the Ag surface, changes in the dielectric constant occur, causing a red-shift of the absorption band. This phenomenon is well described in the literature and is frequently utilized in metal nanoparticle-based optical sensors^39^.

Furthermore, when examining the spectrum of 10 nm AgNPs after the addition of T7wt, a new band at 546 nm becomes apparent, indicating AgNP dispersion destabilization (Fig. 4A, marked with an arrow). As the particles destabilize, the original absorption band diminishes in intensity due to the depletion of stable nanoparticles, and often a secondary band at longer wavelengths emerges due to the formation of aggregates. This result provided additional evidence of the distinct behavior of T7wt and T7Ag-XII in the presence of AgNPs. No AgNPs destabilization was observed in the presence of T7Ag-XII, indirectly suggesting surface interactions (as in the case of capping agents used for stabilization of metal nanoparticle dispersions)^40^.

To further confirm efficient binding of engineered T7Ag-XII to the silver surface, an additional experiment was carried out. Unprotected nanoparticles tend to aggregate and precipitate in the presence of electrolytes. Capping agents, such as various polymers that adsorb on the metal surface, are used to stabilize nanoparticle dispersions. Nanoparticles coated with effective capping agents are less prone to destabilization and precipitation even in the presence of electrolytes^41^. To demonstrate that T7Ag-XII phage adsorbs to the AgNP surface and acts as a stabilizer, an equal amount of 1% NaCl solution was added to the AgNP samples containing T7Ag-XII or T7wt. Dispersion stability was monitored by UV–Vis spectroscopy.

In the case of T7wt phage samples the absorption intensity decreased twice after the addition of NaCl and a distinct new band appeared at approximately 530 nm (see Fig. 4B, black curves), indicating substantial nanoparticle aggregation. The observed effect suggested that there was no interaction between AgNPs and T7wt, and that the Ag surface was not protected by the wild-type phages. Conversely, AgNPs with T7Ag-XII phages were much more stable (red spectra in Fig. 4B), indicating that peptide-presenting T7Ag-XII phages were indeed adsorbed to the Ag surface and positively influenced the AgNP dispersion stability.

### Biofilm eradication using T7 phages and AgNPs

Both T7 phages and AgNPs exhibit antimicrobial activity^18,34,42^. However, the simultaneous action of both agents, particularly recombinant T7 phages displaying AgNP-binding peptides, has not yet been analyzed as an antibiofilm strategy. Therefore, we conducted an experimental analysis of the eradication of *E. coli* biofilms using wild-type T7 phages (wt), recombinant T7Ag-XII phages (displaying the RFEHPAVPRTEM peptide), AgNPs, and T7Ag-XII phages combined with AgNPs. In this experiment, we used 24h *E. coli* biofilms, established in titration plates via growth in Lysogeny Broth (LB) medium. Bacterial biofilm degradation was analyzed at 6 h after the addition of phages and/or AgNPs.

**Figure 5:**
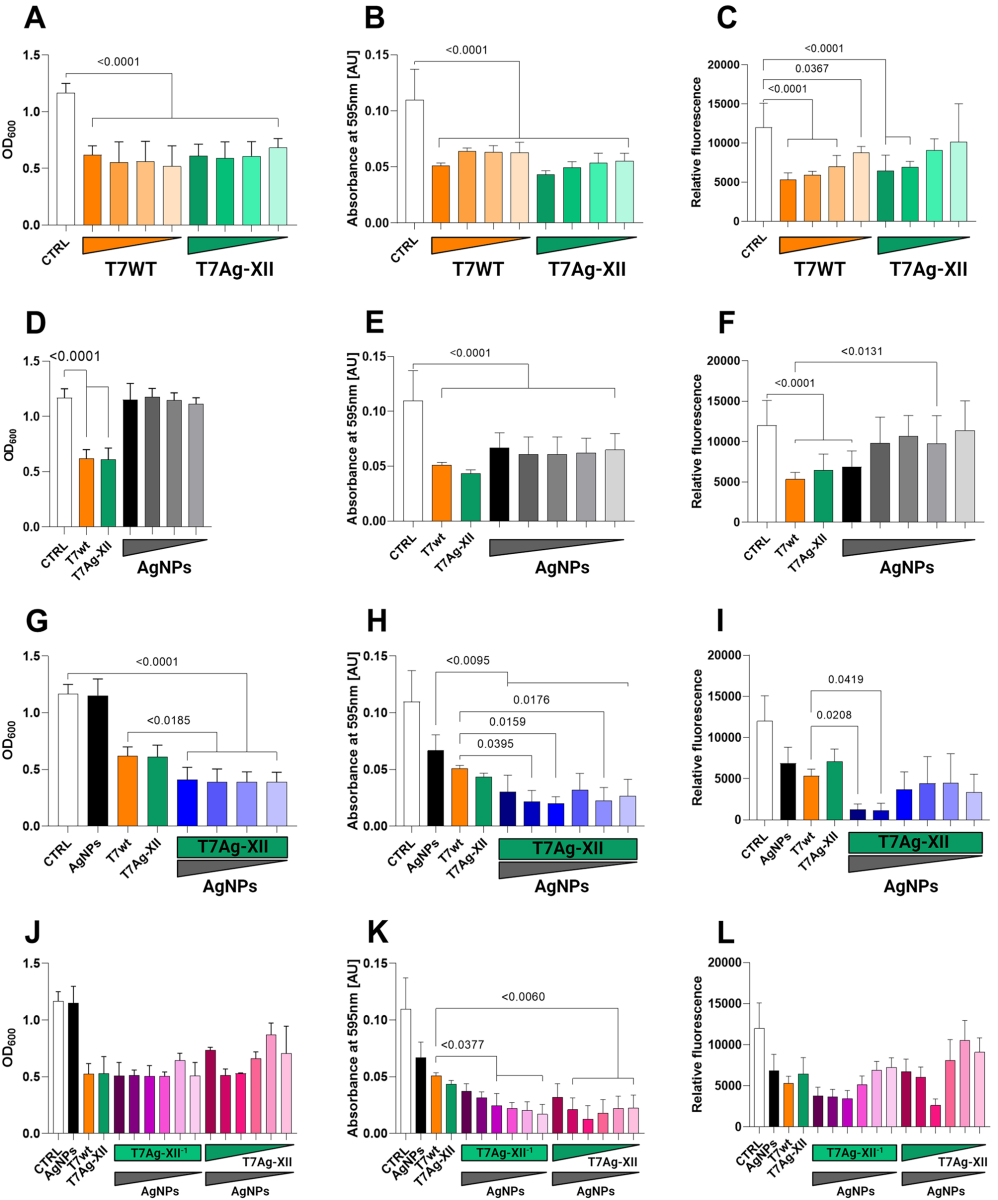
Assessments of biofilms after treatment with T7 phages, AgNPs, or a combination of T7 phages and AgNPs. *Escherichia coli* biofilms were grown for 24 h; incubated for 6 h with T7 and T7Ag-XII phages, AgNPs, or a combination of T7Ag-XII phages with AgNPs; and subjected to analyses of optical density, biofilm biomass, and bacterial viability. The working concentration of phages: T7wt and T7Ag-XII (panels **G**, **H**, and **I**, middle vertical bars) were kept at 1 × 10^9^ pfu/mL; T7Ag-XII^−1^ was kept at 1 × 10^8^ pfu/mL (panels **J**, **K**, **L**, middle vertical bars); the variations in phage working concentrations decreased by an order of magnitude from left to right as indicated by green gradients (panels **A**, **B**, **C**: from 1 × 10^9^ pfu/mL to 1 × 10^6^ pfu/mL; panels **J**, **K**, **L**: from 1 × 10^9^ pfu/mL to 1 × 10^4^ pfu/mL). The working concentration of AgNPs was 1 mg/mL; the variations in AgNP working concentrations decreased by an order of magnitude from left to right as indicated by grey gradients (panels **D**, **G**: from 1 mg/mL to 0.0001 mg/mL; panels **E**, **F**, **H**, **I**, **J**, **K**, **L**: from 1 mg/mL to 0.000001 mg/mL). Panels (**A**, **D**, **G**, and **J**) show measurements of OD_600_; (**B**, **E**, **H**, and **K**) show measurements of biofilm biomass based on crystal violet staining; and (**C**, **F**, **I**, and **L**) show measurements of cell viability based on resazurin staining. Data are shown as mean values of ≥ 3 biological replicates, and standard deviations are represented by error bars. Statistical analysis (one-way ANOVA) was carried out and significant *P*-values are presented. *AgNPs*, Silver nanoparticles; *AU*, Absorbance units; *CTRL*, Biofilm without phages or AgNPs treatment; *OD*_600_, Optical density at 600 nm; *T7Ag-XII*, T7 phages displaying the RFEHPAVPRTEM peptide; *T7wt*, Wild-type T7 phages.

The initial analysis of the antibiofilm activities of T7 and/or AgNPs, based on changes in optical density values of biofilm-forming cultures, is shown in Fig. 5A, D, G, and J. After treatment with either type of T7 phage (wt or Ag-XII), the optical density at 600 nm (OD_600_) values decreased significantly by approximately 50%. There were no differences according to T7 phage type (wt or Ag-XII) or concentration (Fig. 5A). When biofilms were treated with AgNPs alone, there were no decreases in OD_600_ values (Fig. 5D). Notably, the antibiofilm activities of recombinant T7Ag-XII phages (at a concentration of 1 × 10^9^ plaque-forming units [pfu]/mL) combined with various concentrations of AgNPs were significantly higher than the activities of T7wt phages alone or AgNPs alone (Fig. 5G). Lower concentrations of T7Ag-XII phages (1 × 10^8^ pfu/mL) combined with decreasing concentrations of AgNPs did not significantly reduce OD_600_ values in comparison with either type of T7 phages alone (Fig. 5J).

Subsequently, assessments of biofilm biomass (Fig. 5B, E, H, and K) and viability (Fig. 5C, F, I, and L) were conducted. Six hours of treatment with either type of T7 phage (wt or Ag-XII) alone resulted in a significant reduction in biofilm biomass. However, there were no statistically significant differences in efficacy between different phage titers used (Fig. 5B). The use of AgNPs alone seems to be as effective in biomass reduction as the use of T7wt phages alone (Fig. 5E). Importantly, the combined use of T7Ag-XII phages with AgNPs led to a significantly greater reduction of biofilm biomass, compared with the use of T7wt phages alone or AgNPs alone (Fig. 5H). Moreover, the reduction of biofilm biomass was greater when using the combined treatment than when using T7wt phages alone or AgNPs alone, even when the T7Ag-XII phage and AgNP concentrations were reduced (Fig. 5K).

Results of biofilm viability assays confirmed that the T7Ag-XII phages combined with AgNPs had comparatively better antimicrobial efficacy than T7wt phages or AgNPs alone. Bacterial viability significantly decreased after treatment with T7wt or T7Ag-XII phages alone in higher concentrations (Fig. 5C). Exploring the efficacy of AgNPs alone, we observed that only the highest AgNPs concentration was as effective as T7wt phages, and lower AgNP concentrations showed a significant decrease in efficiency compared to T7wt phages (Fig. 5F). Importantly, significant decreases in bacterial viability were observed after combined treatment with T7Ag-XII phages and AgNPs (Fig. 5I). Low concentrations of T7Ag-XII phages combined with AgNPs exhibited a partial capability of reducing biofilm viability; however, the differences with controls were statistically at the same level as when T7wt phages alone were used (Fig. 5L).

In the antibiofilm assays, T7Ag-XII phages and AgNPs were used as a mixture of their suspensions. Therefore, in the final preparations used in the assays, free T7 phages and free AgNPs could be present. To verify whether molecular association between the T7Ag-XII phages and AgNPs is actually necessary for the effects observed, we carried out an important control experiment with the use of T7wt and AgNP mixture, prepared in the same way as in the case of T7Ag-XII and AgNPs. The results clearly showed that T7Ag-XII phages armed with AgNPs have a statistically significant higher antibiofilm efficacy than T7wt phages alone or AgNPs alone (Fig. 1S).

**Figure 6:**
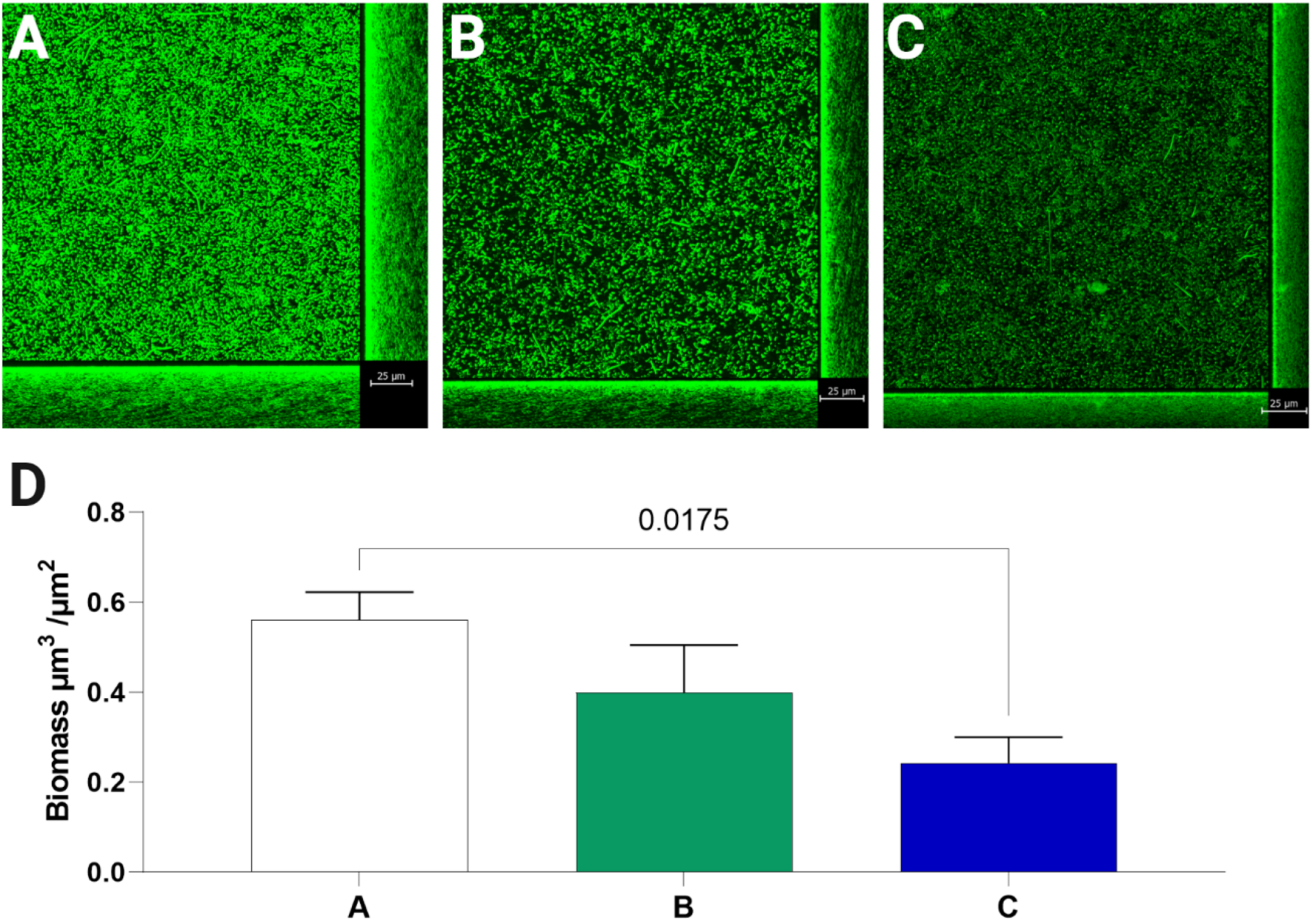
Confocal laser scanning microscopy images of *Escherichia coli* biofilms. Images show *E. coli* biofilms grown for 24 h, then treated for 6 h with phosphate-buffered saline (**A**), T7 phages alone (**B**), or recombinant T7Ag-XII phages combined with AgNPs (**C**). Statistical analysis of biofilm biomass visualized by CLSM was performed with Comstat2 software and significant *P*-value is presented (**D**). Data are shown as mean values of 3 biological replicates, and standard deviations are represented by error bars.

Confocal microscopy analysis confirmed the synergistic antibiofilm activity of recombinant T7 phages and AgNPs (Fig. 6). The greatest biofilm reduction was observed when recombinant T7Ag-XII phages (1 × 10^9^ pfu/mL) were used in combination with AgNPs (0.01 mg/mL).

### Safety analysis of T7Ag-XII phages

**Figure 7:**
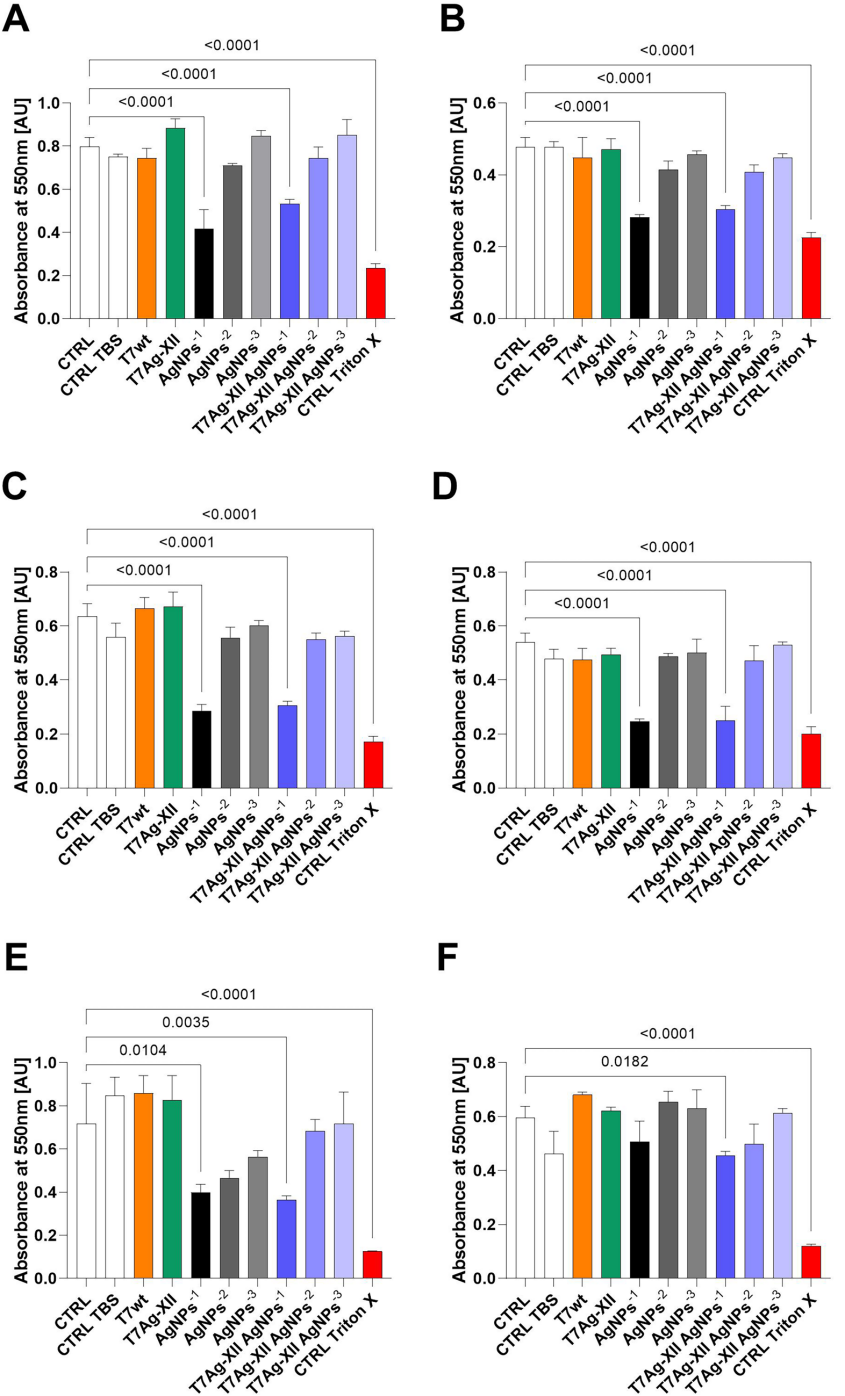
Cytotoxicities of T7 phages and AgNPs to aHDFs and Jurkat E6-1 cells, as determined by MTT assay. Panels show the cytotoxicities of T7wt and T7Ag-XII phages, AgNPs, and T7Ag-XII phages in combination with AgNPs. (**A**, **C**, **E**): aHDFs after 24, 48, and 72 h of incubation, respectively; (**B**, **D**, **F**): Jurkat E6-1 cells after 24, 48, and 72 h of incubation, respectively. The working concentration of T7wt or T7Ag-XII phages was kept at 1 × 10^9^ pfu/mL. The working concentration of AgNPs: AgNPs^−1^ was 0.1 mg/mL; the variations in AgNPs working concentrations decreased by an order of magnitude indicated as AgNPs^−2^, AgNPs^−3^. The working concentration of the combination of T7-XII and AgNPs: T7Ag-XII AgNPs^−1^ was 0.1 mg/mL for AgNPs and 1 × 10^9^ pfu/mL for T7Ag-XII phages; in the variations in the combination of phages and AgNPs, working concentrations of AgNPs decreased by an order of magnitude indicated as T7Ag-XII AgNPs^−1^, T7Ag-XII AgNPs^−2^, T7Ag-XII AgNPs^−3^, and the concentration of phages was constant (1 × 10^9^ pfu/mL). Data are shown as mean values of three biological replicates, and standard deviations are represented by error bars. Statistical analysis (one-way ANOVA) was carried out and significant *P*-values are presented. *AgNPs*, Silver nanoparticles; *aHDFs*, Adult human dermal fibroblasts; *CTRL*, Cells treated with fresh medium; *CTRL TBS*, Cells treated with Tris-buffered saline; *CTRL Triton X*, Cells treated with Triton X; MTT, 3-(4,5-dimethylthiazol-2-yl)-2,5-diphenyltetrazolium bromide; *T7Ag-XII*, T7 phages displaying the RFEHPAVPRTEM peptide; *T7wt*, Wild-type T7 phages.

Both phages and AgNPs reportedly exhibit a low degree of toxicity to eukaryotic cells. However, the presence of phages and/or AgNPs at high concentrations could influence eukaryotic cell viability. Therefore, we conducted a safety analysis using adult human dermal fibroblasts and Jurkat E6-1 cells to determine the cytotoxicities of recombinant T7Ag-XII phages and AgNPs (Fig. 7). These cell lines represent adherent and non-adherent cell models, respectively, and are commonly used in general toxicology and immunology assays.

Cytotoxicity assays revealed that T7 phages were safe for adult human dermal fibroblasts and Jurkat E6-1 cells. However, AgNPs were toxic when used at the highest concentration (0.1 mg/mL). Similarly, T7Ag-XII phages in combination with AgNPs at the highest concentration (0.1 mg/mL) were toxic. We suspect this toxicity resulted from the AgNPs, rather than the T7 phages, because the degree of toxicity was similar to the result of using AgNPs alone. Importantly, treatment with a combination of T7Ag-XII phages and AgNPs was safe for eukaryotic cells when lower concentrations of AgNPs (0.01 and 0.001 mg/mL) were used—these concentrations were most effective for biofilm eradication. Overall, our data suggest that, when used at concentrations that most effectively eradicate bacterial biofilm, T7 phages armed with AgNPs are safe for eukaryotic cells.

## Discussion

According to the most comprehensive analysis of antimicrobial resistance (AMR) burden to date, which provided estimates for 204 countries/territories, 23 bacterial pathogens, and 88 pathogen–drug combinations, an estimated 4.95 million (95% uncertainty interval UI 3.62–6.57) deaths were associated with bacterial AMR, of which 1.27 million (95% UI 0.911–1.71) deaths were directly attributable to bacterial drug resistance over just one year (specifically: 2019)^43^. Those alarming numbers highlight the urgent need for research and development of new effective treatment strategies to tackle antibiotic-resistant infections. The danger associated with bacterial infections increases when they are caused by bacteria that enable the formation of biofilms (i.e., highly structured microbial communities). Biofilm-related infections are presumably responsible for approximately 80% of all infections in humans and animals^44^. Biofilm formation by pathogenic bacteria is regarded as a virulence factor because it protects against host immune response mechanisms and antimicrobial agents^5^.

An alternative to the use of antibiotics for treatment of bacterial infections is phage therapy, which involves the use of bacteria-specific viruses (i.e., phages) to kill bacteria. Phages are easy to isolate and cost-effective; they are also effective in controlling bacteria that have formed biofilms^45^. Although the results of phage therapy have been impressive, this approach has also some limitations. Phages can carry toxin or resistance genes, can physically support a pathological biofilm, or can elicit bacterial resistance to phage infection^46^. The potential for bacterial resistance to phages is particularly challenging with respect to the use of phages as antimicrobial agents. Therefore, in new research efforts phages are being combined with other antimicrobial agents (e.g., antibiotics or enzymes) to improve the efficacy of phage therapy^46,47^.

Metal and organic nanoparticles represent another therapeutic agent that can potentially be administered in combination with phages^48,49^. Nanoparticles, particularly of gold and silver, are reportedly effective in the control of pathogenic bacteria^50^. Thus, we have explored the extent to which combinations of nanoparticles and phages are more effective than either agent alone. To our knowledge, this is the first comprehensive study exploring not only the effectiveness, but also the safety of a therapeutic approach based on synergistic action of recombinant lytic phages and phage-presented nanoparticles. Our research is focused on validating the use of genetically engineered T7 phages that display Ag-binding peptides and are armed with AgNPs to specifically confront the challenge of eradicating bacterial biofilms.

In 1975, Smith published the first report concerning phage display^51^, a technique that involves displaying short peptides on the virions of M13 phages. This display approach is based on genetic engineering of the M13 genome to insert a short nucleotide sequence into the *pIII* gene. The T7 phage can also display peptides on its virion, through genetic engineering of the *gp10B* gene that leads to peptide or protein inclusion in the main gp10 capsid protein^52^. Both M13 and T7 phages are often used to search for and identify novel peptides with specific nanoparticle-binding properties.

Previous studies have shown that genetically engineered M13 phages, exposing recombinant proteins, are capable of not only binding but also synthesizing both the desired peptides and metal nanoparticles on their surface^53–55^. These properties of modified M13 phages are intriguing, particularly because in all previous efforts the desired peptides were synthesized by standard chemical synthesis methods and then used for binding of metal nanoparticles. As demonstrated by this study, such phages that display nanoparticle-binding peptides can also facilitate efforts to control bacterial biofilm. In this study, we have shown that T7 phages armed with AgNPs exhibit greater efficacy in terms of controlling bacterial biofilm, compared with phages or AgNPs alone. This observation may serve as a foundation for novel therapeutic strategies and can facilitate the exploration of new scientific and medical avenues that involve improved phage therapy. We expect that the main feature of such therapy will be the ability to display various proteins/enzymes on different phages and equip those phages with a variety of antimicrobial agents, thereby substantially improving their therapeutic effects.

It should be noted that the application of a combination of phages and NPs has been recognized as a effective strategy for combating both free-growing and biofilm-forming bacterial cells. Previous research has demonstrated the effectiveness of such a mixture against various clinically relevant bacteria. For instance, a φ29-like phage named ϕ44AHJD of the *Podoviridae* family was applied together with AgNPs to destroy a pre-formed biofilm of *S. aureus* Rumba on a glass surface, showing synergistic activity of both components^49^. This effect included not only the dissipation of the biofilm but also lethality toward the biofilm-forming cells. Similarly, phages ZCSE2 and ZCSE6 (of *Myoviridae* and *Siphoviridae* family, respectively) exhibited a synergistic effect with AgNPs against free *Salmonella enterica* cultures^56,57^, as did the mixture of phage LMP3 (of *Podoviridae* family) and AgNPs against liquid cultures of *Listeria monocytogenes*^58^. Additionally, mixing of phage ZCEC5 with AgNPs improved the effectiveness against free *E. coli* O157:H7^59^.

While combinations of phages and nanoscale objects were previously shown to combat bacterial cells or biofilm structures^48,49^, those efforts rarely involved the two components being physically linked to each other. To the best of our knowledge, the only example of such an approach involved conjugating M13—a filamentous phage—to gold nanorods^60^, a procedure requiring chemical modification of the phage surface to facilitate the attachment after each phage propagation procedure. The M13 capsid was modified with N-succinimidyl-S-acetylthiopropionate (SATP) to introduce thiol groups to primary amines. Furthermore, after bioconjugation, CTAB on gold nanorods was replaced by ligand exchange with carboxylated PEG (HS-PEG-COOH). Finally, following excitation of gold nanorods by near-infrared light, the targeted bacterial cells were killed by photothermal ablation. Importantly, phages in that study were used only for attachment to bacterial cells and not as antibacterial agents. Our method relies on NP-binding peptides displayed on structural capsid proteins. Its great advantage is that every generated phage particle is immediately ready to bind NPs, which makes the method much easier to apply. Furthermore, our study is a first report of engineering a lytic phage to bind NPs so that the two components together form a bionanomaterial and synergistically act as part of such a material against biofilms. The T7 phage used in our study is capable of infecting diverse *E. coli* strains as opposed to the above mentioned M13 that naturally adsorbs to only F^+^ *E. coli* strains.

There is some evidence to suggest that AgNPs can inactivate phages or negatively influence phage development in host cells^61,62^. We did not observe such a phenomenon when we mixed our engineered phages with AgNPs. However, the previous reports could explain why we did not observe optimal antibiofilm activity when we used recombinant T7Ag-XII phages armed with the highest concentration of AgNPs. Under our experimental conditions, the use of AgNPs at a high concentration could have negatively influenced the antibiofilm efficacy of T7Ag-XII phages. It may be connected with the many existing possibilities for binding AgNPs to T7 phages with displayed peptides. Examples of different binding combinations that can occur include: every phage-displayed peptide binds one AgNP, two or more peptides from the same phage bind the same AgNP, or peptides from two or more phages bind the same AgNP. In our opinion, in the case of high concentrations of AgNPs, the latter two possibilities are promoted. In those two cases, there are fewer free phages to act or the phages are covered with nanoparticles so tightly that it is more difficult for them to adsorb to bacterial cells. This might have caused the observed decrease in antibiofilm efficacy of recombinant T7Ag-XII phages armed with the highest concentration of AgNPs in our work.

Notably, a decrease in the concentration of nanoparticles led to an increase in phage antibiofilm activity. This finding was unexpected, although it is economically desirable. Lower concentrations of phages and AgNPs with high antibiofilm activity (similar to, or better than, the activities of phages alone or AgNPs alone) incur fewer production costs while reducing the risk of toxicity and the accumulation of AgNPs in tissues and cells.

Phage therapy is considered safe for humans and animals^63^. Phages often exhibit good results in the treatment of bacterial infections, and they do not demonstrate toxicity. Our cytotoxicity results were consistent with previous findings and did not reveal toxic effects associated with phage therapy. Although AgNPs are generally presumed to have low toxicity for humans, they are toxic at high concentrations^50^. Thus, there is a need to consider the potential risk of T7Ag-XII phages armed with AgNPs exhibiting toxic effects on eukaryotic cells. Our observations confirmed that a high concentration of AgNPs affects the viability of eukaryotic cells. However, we found that the use of a lower concentration of AgNPs to arm the engineered T7Ag-XII phages (at a phage concentration with high antibiofilm activity) was safe for eukaryotic cells.

## Conclusions

In conclusion, we have demonstrated a novel strategy to control bacterial infection, based on the combined use of engineered T7 phages and phage-presented AgNPs. We showed that T7Ag-XII phages armed with AgNPs were more effective in terms of eradicating *E. coli* biofilm, compared with phages alone or AgNPs alone. Moreover, significantly better biofilm eradication was observed when lower concentrations of phages and nanoparticles were used. Importantly, our strategy is safe for eukaryotic cells. We expect that our findings will facilitate further exploration of scientific and medical applications involving combination of phages and nanoparticles.

## Methods

### Bacteria and phages

*Escherichia coli* strains C600 (Department of Molecular Virology collection), ER2738 (New England Biolabs), and BLT5403 (Merck™) were used in this study. *E. coli* C600 used for biofilm analyses is a general-purpose host with a closed genome sequence^64^. The C600 strain is free of prophage lambda (λ-) and was previously used in both phage biology research and biofilm assays^65,66^. Cultivation and growth of all bacteria were conducted using Lysogeny Broth (LB), LB agar (1.5% agar), and Top-Agar media (0.7% agar) at 37 °C. Bacteriophages (phages) used in this study were: M13KE (New England Biolabs), recombinant M13 subtypes displaying various peptide sequences within the pIII protein included in Ph.D-12 Phage Display Library (New England Biolabs), T7 wild-type (Department of Molecular Virology collection), and recombinant T7Ag-XII subtypes displaying the selected silver nanoparticle (AgNP)-binding peptide within the gp10B protein (constructed in this study). The phage titers were analyzed with the use of double-layer agar plates; the bottom layer contained around 25 ml of LB agar (1.5%) and the upper layer contained 4 ml of Top-Agar (0.7%) with 200 μL of overnight bacterial culture (*E. coli* ER2738 for M13 and *E. coli* C600 for T7). For analysis of M13 phages used in the biopanning procedure, the bottom agar was supplemented with 100 mM 5-bromo-4-chloro-3-indolyl-beta-D-galactopyranoside (X-gal) and 200 mM isopropyl β-d-1-thiogalactopyranoside (IPTG) in dimethyl formamide (DMF) stock solution (1 mL of solution per 1 L of LB agar).

### AgNPs

AgNPs in the form of silver nanopowder with particle size < 100 nm (Cat. 576,832-5G, Lot# MKBZ5742V) and silver dispersions (0.02 mg/mL in aqueous buffer; 10 nm Cat. 730,785-25 mL, Lot#MKCK8345 and 100 nm Cat. 730,777-25 mL, Lot#MKCN2257) were purchased from Sigma-Aldrich (USA).

### Biopanning procedure

*E. coli* ER2738 strain was inoculated in 10 mL of LB broth medium at 37 °C with vigorous shaking. Ten milligrams of AgNPs were prepared by 6 rapid washes, each using 1 mL of Tris-buffered saline (TBS) plus Tween (TBST; TBS + 0.1% Tween-20). Next, a 100-fold dilution of the Ph.D-12 library was added, and the suspension was gently rocked for 30 min at room temperature (RT). AgNPs with phages were washed 10 times with 1 mL of TBST buffer to remove unbound phages. AgNP-bound phages were eluted with 1 mL of 0.2 M glycine hydrochloride, pH 2.2, by gently rocking the elution mixture for 5 min. The eluate was transferred to a fresh tube and neutralized with 150 μL of 1 M Tris hydrochloride, pH 9.1. Then, a portion of the neutralized eluate was used for full-plate titration on X-gal/IPTG plates. The remaining neutralized eluate was added to a culture of *E. coli* ER2738 for propagation. After 5 h of incubation, the culture of *E. coli* ER2738 was centrifuged at 12,000 g for 10 min at 4 °C to separate bacteria from phages. The supernatant was transferred to a fresh tube, mixed with 20% polyethylene glycol, and incubated overnight at 4 °C. Subsequently, the precipitate was centrifuged at 12,000 g for 15 min at 4 °C. The resulting pellet was resuspended in 1 mL of TBS and centrifuged again at 14,000 g for 5 min at 4 °C. The phage concentration was determined by titration on X-gal/IPTG plates. The remaining phages were subjected to another round of biopanning. After three rounds of biopanning, phages from single plaques were separately propagated for single-stranded DNA isolation and sequencing analysis.

### AgNP-binding efficacy

Binding efficacy was calculated as the output–input ratio, in accordance with the method of Sano and Shiba^33^. The output phage number is the titer of the eluted AgNP-binding phages, while the input phage number is the total number of peptide-displaying phages used in the AgNP-binding assay. For each test phage, 10 mg of AgNPs were washed with TBST, as described in the biopanning procedure. Next, a suspension of approximately 10^9^ plaque-forming units (pfu)/mL of the test phage (i.e., input phage) was added to the AgNPs and incubated with gentle rocking for 30 min at RT. After washing and elution steps, the phage concentration was determined as described in the biopanning procedure. This phage preparation comprised the output phage, and it was subjected to titration on double-agar X-gal/IPTG plates. M13KE phage with the wild-type pIII protein was used as a control.

### Construction of recombinant T7 phages displaying selected Ag-XII peptide

The commercially synthesized oligonucleotides T7Ag-XII_up (AATTCT**GGTGGAGGTCGGTTTGAGCATCCTGCGGTGCCTCGTACGGAGATG**TAAA) and T7Ag-XII_down (AGCTTTTA**CATCTCCGTACGAGGCACCGCAGGATGCTCAAACCGACCTCCACC**AG) were mixed together with 10× Buffer O (Thermo Scientific™) at a ratio of 1:1:2. In the above sequences, the portion of each oligonucleotide that forms a single-stranded overhang is underlined, whereas the regions that encode peptides are bolded. The annealing mixture was heated in an Eppendorf Mastercycler to 95 °C for 5 min. It was then subjected to 70 heating cycles of 30 s each; the first cycle was set at 95 °C, and each subsequent cycle used a temperature 1 °C lower than the previous cycle. After annealing, DNA fragments were phosphorylated with T4 PNK (Thermo Scientific™) and ligated with T4 DNA ligase (Thermo Scientific™) into T7Select *Eco*RI/*Hin*dIII Vector Arms using the T7Select Cloning Kit (Merck™) in accordance with the manufacturers’ instructions.

Each reaction containing the assembled genomic DNA of a recombinant phage was mixed with T7 Packaging Extract (Merck™) at a ratio of 1:5 (phage: T7 Packaging Extract). The mixture was incubated at RT for 2 h. Next, the titers of the resulting recombinant phages were determined on double-agar plates containing *E. coli* BLT5403 (Merck™). Individual plaques were selected for the amplification of individual phage clones. The resulting lysates were used as templates for polymerase chain reaction with the primers T7_seqF (CGCTCGCCGTGCTAACTTCC) and T7_seqR (GCATCTGCGTTAGCGTCACC). The resultant DNA fragments were sequenced to identify correct T7Ag-XII clones.

### UV − Vis absorption spectroscopy measurements

Colorimetric analyses were performed on single-channel absorbance, fluorescence, and luminescence Synergy HT (BioTEK) microplate reader using Corning® UV-transparent 96-well plates (Sigma-Aldrich). Spectra were recorded in the 200–900 nm range. In the first experiment, 100 µL of each AgNP dispersion and 100 µL of T7wt or T7Ag-XII at concentrations of 1 × 10^9^ pfu/mL were mixed and incubated for 30 min at RT. In the second approach, 50 µL of 10 nm AgNPs was mixed with 150 µL of water (control), T7wt, or T7Ag-XII (at concentrations of 1 × 10^9^ pfu/mL), and incubated for 30 min at RT. Subsequently, 40 µL of 1% NaCl water solution was added. All analyses were carried out in three replicates, and representative curves were presented.

### Biofilm assays

Biofilm biomass and viability after treatment with T7 phages and/or AgNPs were analyzed in accordance with the methods of Latimer et al.^67^ and Skogman et al.^68^, with some modifications. An overnight culture of *E. coli* strain C600 was diluted 1:100 in fresh LB broth medium, then aliquoted into 96-well microplates with 200 µL of bacterial culture per well. After 24 h of incubation at 37 °C, mature biofilm was washed 5 times with phosphate-buffered saline (PBS). After the last washing, 180 µL of PBS was added to each biofilm. Next, 20 µL of various types of T7 phages and/or AgNPs were added. Analyses were performed 6 h after the addition of the test agents.

Negative control biofilms were treated with PBS alone. LB lysates of T7 wild-type (T7wt) and T7Ag-XII phages at an initial concentration of 1 × 10^10^ pfu/mL, and a suspension of AgNPs in water at an initial concentration of 10 mg/mL, were used as controls for the efficacy of the test agents alone. Serial decimal dilutions in PBS were prepared as needed. The combination of T7Ag-XII with AgNPs was prepared by incubating T7Ag-XII with AgNPs for 30 min at RT. Serial decimal dilutions in PBS were prepared as needed.

The optical density of each treated biofilm was measured (λ = 600 nm) using a Sunrise plate reader (Tecan) directly before biofilm biomass and viability assays. To measure the viabilities of cells in treated biofilms, the media was removed from microplates; all wells were washed twice with PBS buffer, filled with 200 µL of resazurin solution (0.2 mM), and incubated for 30 min at 37 ℃. Subsequently, the contents of the wells were transferred to a black 96-well microplate, and fluorescence (λ_ex_ = 520 nm; λ_em_ = 590 nm) was measured using a Synergy H1 multimode plate reader (BioTek). Next, biofilm biomass was measured after wells in the initial microplate had been washed with PBS buffer to remove residual resazurin. First, each biofilm was fixed with methanol for 10 min. The methanol was then removed; biofilm biomass was stained with crystal violet (1% w/v) by incubation for 20 min at RT. Subsequently, the crystal violet was removed and microplates were washed with running water, then allowed to dry at RT. Next, a solution of acetone and ethanol (at a ratio of 2:8) was applied by incubation for 10 min at 37 ℃ with shaking (100 rpm) to remove excess stain. Finally, absorbance was measured (λ = 595 nm) using a Synergy H1 multimode plate reader (BioTek).

### Scanning confocal laser microscopy

Scanning confocal laser microscopy was used to visualize biofilms after treatment with wild-type T7 phages or T7Ag-XII phages armed with AgNPs. For this purpose, suspensions of *E. coli* strain C600 (1 × 10^8^ cells/mL) were prepared in LB broth. Then, 3 mL aliquots of the bacterial suspensions were added to glass-bottom confocal dishes (35 mm diameter, 20 mm glass bottom) (VWR), and bacterial growth was continued at 37 °C for 24 h. Next, 300 µL of wild-type T7 phages or recombinant T7Ag-XII phages combined with AgNPs were added. Analyses were conducted 6 h after the addition of the test agents. Negative control biofilms were formed by *E. coli* in the absence of AgNPs and T7 phages. All experiments were carried out in 3 biological replicates.

After incubation, the medium was removed and each biofilm was washed 3 times with 10 mM MgSO_4_. Then, the biofilm was stained by adding 3 mL of acridine orange solution (10 µg/mL in 10 mM MgSO_4_) and incubating for 30 min at RT. Subsequently, the biofilm was rinsed twice with 10 mM MgSO_4_. Confocal microscopy was conducted using an Eclipse Ti (A1) microscope (Nikon) equipped with a 60 × , 1.4-numerical aperture oil immersion phase-contrast lens. An argon laser with a maximum emission line at 488 nm was used as the excitation source. Horizontal optical thin sections were collected at 0.21-µm intervals from the outer surface of the biofilm to the bottom of the glass plate. These images were captured by NIS-ELEMENTS interactive software (Nikon) and three-dimensional reconstructions were created.

Confocal images of biofilms were analyzed using COMSTAT2 software^69^ (http://www.COMSTAT2.dk, Lyngby, Denmark). Biofilm biomass (µm^3^/µm^2^) was quantified as the volume of all voxels, above a threshold calculated as per Otsu's method^70^, that contains biomass divided by the total image area.

### Cell lines and cytotoxicity assays

Primary adult human dermal fibroblasts (American Type Culture Collection [ATCC]: PCS-201–012™) and human Jurkat T lymphoblasts, clone E6-1 (ATCC: TIB-152™) were used for cytotoxicity assays. Fibroblasts were cultured in Fibroblast Basal Medium (ATCC: PCS-201–030™), supplemented with Fibroblast Growth Kit-Low Serum (ATCC: PCS-201–041™), at 37 °C with 5% CO_2_. Jurkat cells were cultured in Roswell Park Memorial Institute (RPMI)-1640 medium (ATCC: 30–2001), supplemented with 10% fetal bovine serum (Biowest) (v/v), at 37 °C with 5% CO_2_.

For analysis of cytotoxicity, the 3-(4,5-dimethylthiazol-2-yl)-2,5-diphenyltetrazolium bromide (MTT) assay was used. In this assay, the reduction of a yellow tetrazolium salt to purple formazan crystals by metabolically active cells is regarded as an indicator of cell viability, proliferation, and cytotoxicity. The Cell Proliferation Kit I (MTT) (Roche) was used for MTT assays, in accordance with the manufacturer’s instructions. Briefly, 80 µL/well (1 × 10^4^ cells) of a suspension of fibroblasts or Jurkat cells in the appropriate medium (described above) were seeded in 96-well, flat-bottom microplates (tissue culture grade); this was followed by the addition of 20 µL of phages and/or AgNPs. The cells and test agents were incubated for 24, 48, or 72 h at 37 °C with 5% CO_2_. At each time point, 10 μL per well of the MTT labeling reagent (final concentration, 0.5 mg/mL) was added to a subset of wells, and the microplates were incubated for 4 h at 37 °C with 5% CO_2_. Next, 100 μL of solubilization solution was added to each well, and the plates were incubated overnight at 37 °C with 5% CO_2_. Formazan production was measured at 550 nm using a Sunrise microplate reader (Tecan). In control reactions, fibroblasts or Jurkat cells were treated with Triton-X100, TBS, or a fresh aliquot of the appropriate medium (described above). Additionally, before fibroblasts or Jurkat cells were seeded in microplates, their cell viability was assessed by trypan blue staining.

### Supplementary Information

Supplementary Information.

## Data Availability

The raw data required to reproduce these findings are available to download from P. Golec. The processed data required to reproduce these findings are available to download from P. Golec.
