# Supplementary material for: An effective antibiofilm strategy based on bacteriophages armed with silver nanoparticles
Source: Sci Rep. 2024 Apr 20;14:9088. doi: 10.1038/s41598-024-59866-y (PMC11032367; doi:10.1038/s41598-024-59866-y)
Supplement: Supplementary file 1 — Supplementary Information. [file 41598_2024_59866_MOESM1_ESM.pdf]

Supplementary Information

An effective antibiofilm strategy based on bacteriophages armed with silver nanoparticles

Mateusz Szymczak[1], Jarosław A. Pankowski[1,2], Agnieszka Kwiatek[1], Bartłomiej Grygorcewicz[1], Joanna Karczewska-Golec[1], Kamila Sadowska[3] and Piotr Golec[1,*]

[1] Department of Molecular Virology, Institute of Microbiology, Faculty of Biology, University of Warsaw, Miecznikowa 1, 02-096 Warsaw, Poland

[2] Dioscuri Centre for Physics and Chemistry of Bacteria, Institute of Physical Chemistry, Polish Academy of Sciences, Kasprzaka 44/52, 01-224 Warsaw, Poland

[3] Nalecz Institute of Biocybernetics and Biomedical Engineering, Polish Academy of Sciences, Ks. Trojdena 4, 02-109 Warsaw, Poland

* Corresponding author: Piotr Golec
University of Warsaw
Faculty of Biology,
Institute of Microbiology
Department of Molecular Virology,
Miecznikowa 1, 02-096 Warsaw, Poland
Tel: 48 225541421
Fax: 48 225541402
E-mail: piotr.golec@uw.edu.pl

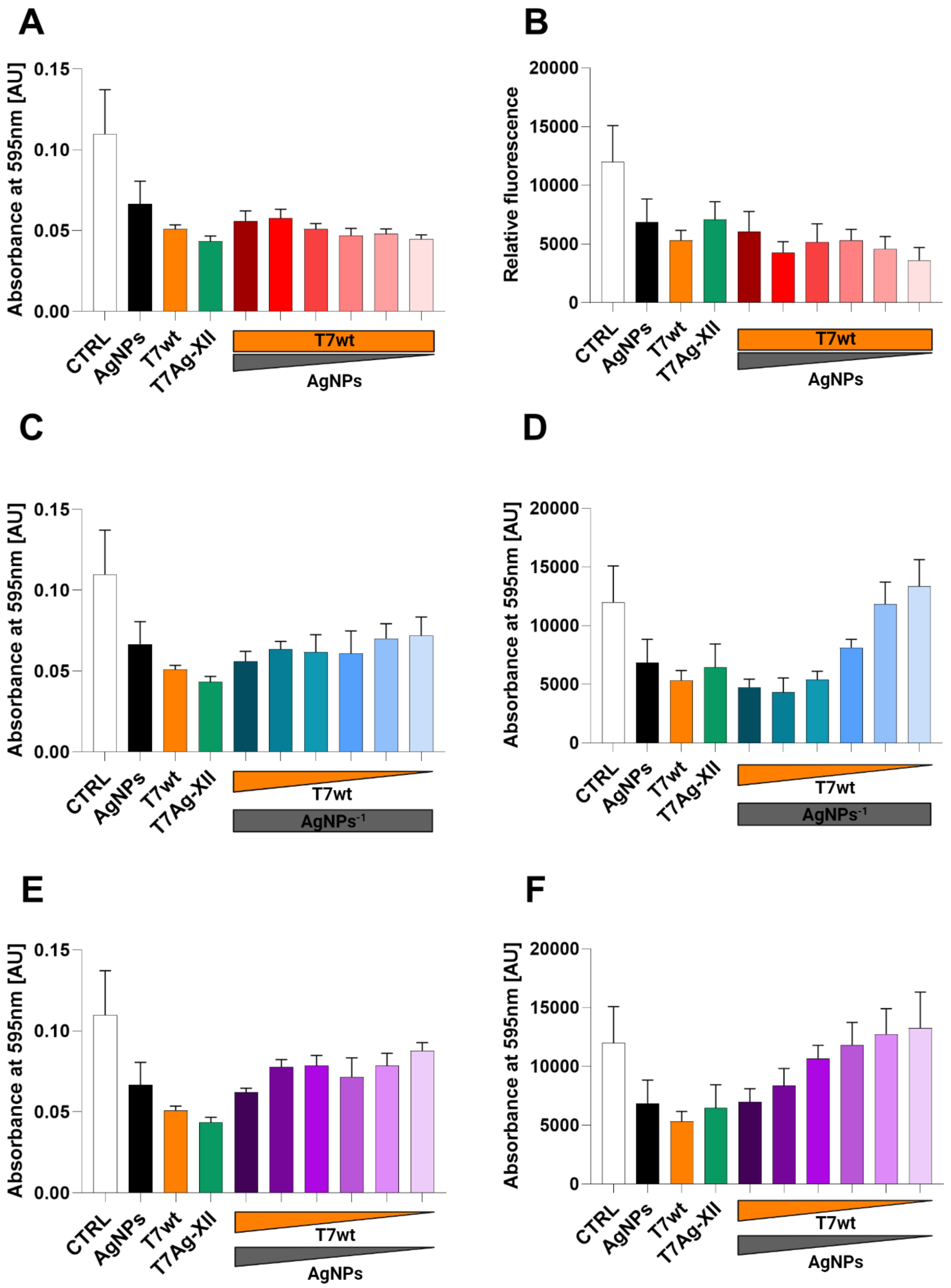

Figure 1S. Assessments of biofilms after treatment with a combination of T7wt phages and AgNPs. *Escherichia coli* biofilms were grown for 24 hours; incubated for 6 hours with

T7wt and T7Ag-XII phages, or a combination of T7wt phages mixed with AgNPs; and subjected to analyses of biofilm biomass and bacterial viability. The working concentration of phages: T7wt and T7Ag-XII were kept at 1×10^9 pfu/mL; the variations in T7wt phage working concentrations decreased by an order of magnitude from left to right as indicated by orange gradients (panels C, D, E and F: from 1×10^9 pfu/mL to 1×10^4 pfu/mL). The working concentration of AgNPs was 1 mg/mL; the variations in AgNP working concentrations decreased by an order of magnitude from left to right as indicated by grey gradients (panels A, B, E and F: from 1 mg/mL to 0. 000001 mg/mL). Panels A, C, and E show measurements of biofilm biomass based on crystal violet staining; and B, D, and F show measurements of cell viability based on resazurin staining. Data are shown as mean values of ≥3 biological replicates. Statistical analysis (one-way ANOVA) was carried out. There is no significantly higher efficiency of T7wt phages mixed with AgNPs in comparison with AgNPs alone or T7wt alone. Abbreviations: AgNPs, silver nanoparticles; AU, absorbance units; CTRL, biofilm without phages or AgNPs treatment; T7Ag-XII, T7 phages displaying the RFEHPAVPRTEM peptide; T7wt, wild-type T7 phages.

Table S1. Amino acid sequences of AgNP-binding peptides displayed within the pIII protein in M13 phages after three rounds of biopanning.

Clone name	Sequence	pI$^{\alpha}$	MW [Da]
Ag-I	SWNDPFQPQYYS	3.80	1531.60
Ag-XI	LIWGSNMNLLPI	5.52	1370.67
Ag-XII	RFEHPAVPRTEM	6.76	1469.68
Ag-XIV	LPYSARTFFSEA	6.00	1388.54
Ag-XV	SYARVDLPEHIH	5.94	1436.59
Ag-XVII	PGDGTLSRYNNY	6.26	1356.41
Ag-XVIII	PWQQRPPDYHTD	5.22	1539.63

$^{\alpha}$Isoelectric point (pI) and molecular weight (MW) values were calculated using the Compute pI/MW tool available at https://www.expasy.org/resources/compute-pi-mw.
